# Nursing Care Ethical Implications Regarding Chronic Patients at Hospital Discharge

**DOI:** 10.3390/healthcare8020167

**Published:** 2020-06-11

**Authors:** Valle Coronado-Vázquez, Carlota Canet-Fajas, María Valle Ramírez-Durán, Juan Gómez-Salgado, José Miguel Robles-Romero, Javier Fagundo-Rivera, Macarena Romero-Martín

**Affiliations:** 1 Department of Nursing, Catholic University of Ávila, 05005 Ávila, Spain; mvcoronado@msn.com (V.C.-V.); mvalle.ramirez@ucavila.es (M.V.R.-D.); 2 Primary Care Research Unit, Red de Investigación en Actividades Preventivas y Promoción de la Salud (RedIAPP), University of Zaragoza, 50009 Zaragoza, Spain; 3 Delicias Sur Community Health Center, Aragon Health Service, 50009 Zaragoza, Spain; carlotacanet@gmail.com; 4 Department of Sociology, Social Work and Public Health, Faculty of Labour Sciences, University of Huelva, 21007 Huelva, Spain; 5 Safety and Health Postgraduate Programme, Espiritu Santo University, Guayaquil 092301, Ecuador; 6 Department of Nursing, Faculty of Nursing, University of Huelva, 21071 Huelva, Spain; macarena.romero@denf.uhu.es; 7 Andalusian Health Service, Health Sciences Doctorate School, University of Huelva, 21007 Huelva, Spain; javier.fagundo308@alu.uhu.es

**Keywords:** ethics, nursing care, chronic patient, hospital discharge

## Abstract

Mortality rates among pluripathological patients are significantly higher in the hospital setting, with advanced age and dependence on certain vital functions the main clinical aspects. Other features involved in the care, such as the loss of autonomy and social problems, have important ethical implications. The aim of this article is to analyze the health problems and the functional and social situation of chronic patients after hospital admission in order to determine their care needs and the ethical implications these might have. For this, a cross-sectional descriptive study is being carried out with a sample of 111 chronic pluripathological patients admitted to the internal medicine service and discharged later. Overall, 96.6% of the patients in the sample were dependent, 91.7% had social problems or were at social risk and 36.9% had cognitive impairment. Among dependent patients, 59.4% had social problems (*p* = 0.029), 19.2% lived alone (*p* = 0.13), and in 73.3% of cases the housing was inadequate (*p* = 0.47). Among those with cognitive impairment, 79.5% of patients had social problems (*p* = 0.001), and 10.3% lived alone (*p* = 0.038). The results of the study confirm the presence of dependence and social problems at hospital discharge in a high proportion of chronic patients. Planning their care can lead to ethical conflicts related to the use of information technologies, which are destined to promote the patients’ autonomy, and to the social problems associated with the illness.

## 1. Introduction

The rapid increase in patients with chronic illnesses in societies with a high percentage of aging population is forcing change in healthcare models and healthcare guidance towards new needs [1]. 

Chronic illnesses are associated with factors such as age, sex, and level of studies, being more common among the elderly, women and populations with a lower level of studies [2].

Multimorbidity ends up affecting the patients’ quality of life, generating dependence and disability [3], so early implementation of healthcare and monitoring of the elderly may help prevent the progression to dependence [4]. The indexes to determine multimorbidity have proven to be highly heterogeneous. Pluripathology and multimorbidity are considered present when two or more chronic illnesses collide that produce a special clinical fragility and the progressive deterioration of the patient [5]. This rather simplistic definition is subject to questioning when considering that most elderly people have various pathological processes. All this suggests that the correlation between social, psychological and quality of life factors should be considered [6].

This study uses Ollero’s definition of the pluripathological patient as a patient that presents illnesses included in two or more clinical categories proven to be related to increased functional limitations, mortality, and use of resources [7,8]. In a study developed in Andalusia, Spain, the prevalence of pluripathological patients among the general population was 1.4%, rising to around 5% in those aged 64. In patients attended by general internal medicine services, this figure reaches 30%, and is close to 60% regarding medical services for chronic patients [7,8].

In primary care, 94% of pluripathological patients are polymedicated, 34% have a Barthel score of less than 60, 37% of them suffer cognitive impairment, and more than 60% need the help of a carer [9,10].

Mortality rates among pluripathological patients have been recently determined as significantly higher in the hospital setting, regardless of the causes of hospitalization. The factors associated with negative life prognosis include old age and a worsening functional situation. These patients also deteriorate further during hospitalization than those who do not present pluripathology [11]. 

Nursing professionals play an important role in managing chronic illnesses in primary care, mainly in planning the healthcare to be given and in managing the resources these patients require [12]. In addition, new technologies can help change the way these patients are cared for at home, shifting to high-quality care at a lower cost [13]. The use of information and communications technologies (ICT) in nursing care allows for integration of technological competencies in the nursing practice and the commitment with an efficient practice [14]. This happens with chronic diseases such as diabetes or hypertension, where ICTs facilitate patients’ empowerment and independence regarding their own care [15].

The work developed by nursing with patients with chronic diseases must be oriented towards promoting autonomy and self-care, understanding autonomy is not an individual activity but one that implies a responsibility shared among patients, professionals and care givers [16].

In recent years, a care model has been widespread to try, train and empower patients with the aim of making them the key actors in their own care, an approach that has been called “patient-centered care”. Although there are many definitions of “patient-centered care”, it has been broadly conceived as “providing care that is respectful of and responsive to individual patient preferences, needs, and values, ensuring that patient values guide all clinical decisions” [17]. This concept is framed within the “person-centered care” concept, as it considers that planning care strategies not only affects patients’ health issues, but also their abilities to cope with them [18].

Nurses encounter several ethical conflicts during their care practice with chronic patients, such as autonomy limits when self-care practices are promoted or their own sense of responsibility towards their care. The solution to these ethical conflicts may arise from the promotion of autonomy as a responsible practice where who will be in charge of the care is decided, as well as how and which part of the care that person will perform [19]; or, from a relational perspective of nursing care, where autonomy is promoted from a more global approach in which there is an interaction between patients’ values and preferences, nursing knowledge and abilities, and the social context of the patient [20].

Another issue is whether there is sufficient social support to cover patients’ non-clinical needs; their autonomy may be hindered due to dependence on this social network. As a result, an intervention towards patients becoming actors in their care must be performed fairly so that the most dependent ones and those with less probabilities of becoming active receive greater attention and further resources [21].

There are different dimensions involved in the care of pluripathological patients such as the loss of autonomy and social problems, which pose important ethical implications.

The objective of this study was to analyze the health problems and functional and social situation of chronic pluripathological patients after hospital admission, in order to determine the caring needs and their ethical implications.

## 2. Materials and Methods 

### 2.1. Study Design and Setting

A cross-sectional descriptive study was conducted on a sample of 111 patients diagnosed with pluripathology and who were discharged after admission to the internal medicine service of a Huelva hospital (Spain).

The patients were recruited by consecutive sampling if they presented illnesses included in two or more pluripathology diagnostic categories. Patients who were referred to a nursing home after hospital discharge were excluded.

Data were collected through a structured questionnaire that included variables such as the age of the patient at discharge, sex, and the number and type of chronic illnesses at the time of inclusion in the study.

The degree of social support received by the patient was measured according to the Gijón Socio-Family Evaluation Scale [22]. This is a heteroadministered test that assesses family and social risk, and which consists of five items (family, economic, housing, relational situation, and social support). Each item may have a scoring between 1 (ideal social situation) and 5 (established social problems). A score lower than or equal to 9 is considered normal or low social risk; from 10 to 15 points, the risk is intermediate; and a score higher than or equal to 16 points corresponds to established social problems.

Patient dependence levels were measured using the Barthel scale, which calculates the degree of patient autonomy when performing basic daily tasks [23]. This assessment is based on scores on a scale of 0 to 100 (from total dependence to total independence).

The level of cognitive impairment was measured using the Pfeiffer’s questionnaire [24].

### 2.2. Analysis

The data were analyzed using the SPSS 17.0 statistical package (IBM: Armonk, NY, USA). The characteristics of the sample were analyzed by calculating the arithmetic mean and standard deviation for the quantitative variables, and the absolute and relative frequencies for the qualitative variables.

### 2.3. Ethical Consideration

The study protocol was approved by the Huelva Ethics Committee with registration number PI 042/14. After approval, the researchers provided some oral information to the participants including the goals and objectives of the study and the confidentiality and anonymity of the data; the participants were free to withdraw from the study at any time. An informed consent was obtained individually.

## 3. Results

111 patients who met the inclusion criteria were included in the study.

The mean age of the participants was 78.7 (SD 8.5) years. Other characteristics of the study participants are shown in Table 1.

The mean admission time was 11.4 days (SD 5.9). The most frequent diagnoses at discharge were related to the circulatory system (59.9%), respiratory system (20.8%), and nervous system (4.2%). The Barthel index mean was 51 (SD 29.5) and the Gijón scale mean was 14.4 (SD 2.9).

In total, 96.6% of the patients in the sample were dependent, 91.7% had social problems or were at social risk and 36.9% had cognitive impairment. 

Among dependent patients, 59.4% had social problems (*p* = 0.029), 19.2% lived alone (*p* = 0.13), and in 73.3% of the cases the housing was inadequate (*p* = 0.47). Among those with cognitive impairment, 79.5% of patients had social problems (*p* = 0.001), and 10.3% lived alone (*p* = 0.038). See Table 2.

## 4. Discussion

The results of the study show a high percentage of chronic pluripathological patients in situations of dependence, cognitive impairment, and social problems. This has important ethical implications for the community care these patients need.

Dependence is frequent among the elderly with pluripathology. The findings of this work coincide with another study [25] that describes a Barthel’s mean score of 52.04 (SD 33.16), which was lower than the scores found in other studies [26]. In these patients, promoting a more active role in their own care, acquiring greater responsibility and autonomy in decision-making, and the capacity to carry out these decisions are of key importance [27]. Nursing professionals can help promote self-care and patient training through healthcare education measures. These interventions should be individualized according to the values and preferences of the patients, through shared decision-making [28], as well as on the basis of the available resources. However, the degree of responsibility and participation of the patients in their own care differs depending on the type and complexity of their illness, their degree of dependence, and their social-health situation. 

Healthcare for the chronically ill should be planned considering the disability and dependence that accompanies such illness, implementing social support measures and using information and communication technologies, which have been shown to improve health outcomes [29,30]. It should be borne in mind that applying these technologies may give rise to ethical conflicts in certain circumstances such as the use of home telemonitoring in patients with cognitive impairment, due to the interference this may cause in people’s privacy [31].

In this study, the mean score in the Gijon scale is higher than the one described for a sample of pluripathological patients in primary care [32], which indicates higher social risk. In addition, like other social determinants of health such as education or housing conditions, loneliness is also related to multimorbidity [33].

In this study, dependent and cognitively impaired patients have shown problems related to social support deficits, housing conditions, and economic income. The chronification of illnesses may be conditioned by social factors, so an integrated healthcare model that harmonizes the actions of social and health services is needed [34]. This would be a more equitable model by preventing avoidable complications of the illness and reducing system inefficiencies by decreasing test duplicates, unnecessary hospitalizations, and the use of other healthcare resources [35,36].

An equitable distribution of healthcare resources means allocating special resources for those people who need them most [37,38]. For this, the stratification of chronic patients is useful, which aims to organize health services according to the patients’ needs and make them more efficient by establishing positive discrimination on the basis of these patients’ vulnerability; this would imply fragile patients receiving protection from the healthcare system [39]. When this stratification is carried out by only considering the clinical and demographic variables but without taking into account the social factors, it can become biased by discriminating against those who have worse social conditions, as compared to those with greater clinical complexity but with economic resources and social support.

Comprehensive healthcare is one of the components of "person-centered care” that aims to help patients become more active and promote the role of primary care in coordinating healthcare [40]. From an ethical point of view, patient-centered care implies a deep respect for people's dignity, and so it is carried out by informing, listening, respecting, and involving the patients in the decisions that affect their health [41].

The findings of this study have implications for scientific-evidence-based practice as they highlight the importance of giving humanized care where communication abilities, responsibility and ethical commitment are enhanced. This shall be achieved by developing quality care based on the available evidence from a holistic approach towards patients and their contexts.

One of the main limitations of this study is the sample size. The highly homogenous distribution of some variables can be explained by the sample recruited at hospital discharge. The hospital covered an area whose residents were mainly elderly people with high comorbidity.

## 5. Conclusions

Most pluripathological patients have some degree of dependence at hospital discharge, presenting problems such as lack of social support and inadequate housing conditions.

This poses a challenge for nursing professionals, who must take into consideration the disability and dependence of patients when planning their healthcare and use information technologies to promote their autonomy while respecting their dignity as individuals. Ethical care requires dialogue with professionals from other disciplines in order to improve the social conditions of these patients.

## Figures and Tables

**Table 1:** Sample characteristics.

**Sex, men**	**(%)** 56.8
**Medication, Mean**	**(SD)** 9.1 (3.1)
**Functional situation (Barthel index)**	***n* (%)**
Independent	7 (6.3)
Moderate dependence	35 (31.5)
Severe dependence	47 (42.3)
Total dependence	22 (19.8)
**Social risk (Gijón scale)**	***n* (%)**
Social problem	61 (56.5)
Social risk	38 (35.2)
Good social situation	9 (8.3)
**Cognitive impairment (Pfeiffer)**	***n* (%)**
No impairment	69 (62.2)
Cognitive impairment established	41 (36.9)

**Table 2:** Relationship between cognitive impairment and loneliness and social risk.

Cognitive Impairment *n* (%)	Yes	No	*p*
**Loneliness**			0.038
Lives alone	4 (10.3)	18 (27.3)	
Lives accompanied	35 (89.7)	48 (72.7)	
**Social risk**			0.001
Positive social situation	1 (2.6)	8 (11.8)	
Social risk exists	7 (17.9)	31 (45.6)	
Social problem exists	31 (79.5)	29 (42.6)	
